# Supplementary material for: The Systems Biology Research Tool: evolvable open-source software
Source: BMC Syst Biol. 2008 Jun 29;2:55. doi: 10.1186/1752-0509-2-55 (PMC2446383; doi:10.1186/1752-0509-2-55)
Supplement: Additional file 1 — SBRT Archive. An archive of the current version of the Systems Biology Research Tool. [file 1752-0509-2-55-S1.zip › sbrt-1.4.0/doc/developers_guide/api/sbrt/kernel/math/geom/comp/class-use/CdHarComputer.Results.html]

Uses of Class sbrt.kernel.math.geom.comp.CdHarComputer.Results


|  |  |  |
| --- | --- | --- |
| **Overview** \| **Package** \| **Class** \| **Use** \| **Tree** \| **Deprecated** \| **Index** \| **Help** | |  |
| PREV   NEXT | **FRAMES**    **NO FRAMES**     **All Classes** |  |


---


## **Uses of Class sbrt.kernel.math.geom.comp.CdHarComputer.Results**

| Packages that use CdHarComputer.Results | |
| --- | --- |
| **sbrt.kernel.math.geom.comp** | Provides classes and interfaces for performing geometry-related computations. |
| **sbrt.kernel.math.geom.proc** | Provides classes and interfaces for performing geometry-related processes. |

Uses of CdHarComputer.Results in sbrt.kernel.math.geom.comp

| Methods in sbrt.kernel.math.geom.comp that return CdHarComputer.Results | |
| --- | --- |
| `CdHarComputer.Results` | `CdHarComputer.next()`             Computes and returns the next random interior point of the convex polytope. |
| `CdHarComputer.Results` | `CdHarComputer.next(int totalPoints)`             Computes the specified number of random interior points, and returns the last one computed. |
| `CdHarComputer.Results` | `CdHarComputer.run(java.util.Map<? extends V,java.lang.Double> startPoint)`             Computes and returns a random interior point of the convex polytope starting from the provided point. |

Uses of CdHarComputer.Results in sbrt.kernel.math.geom.proc

| Method parameters in sbrt.kernel.math.geom.proc with type arguments of type CdHarComputer.Results | |
| --- | --- |
| `void` | `CdHarProcess.run(ResultsManager<CdHarComputer.Results> resultsManager)`             Adds random points to the provided results manager. |

---


|  |  |  |
| --- | --- | --- |
| **Overview** \| **Package** \| **Class** \| **Use** \| **Tree** \| **Deprecated** \| **Index** \| **Help** | |  |
| PREV   NEXT | **FRAMES**    **NO FRAMES**     **All Classes** |  |


---
